# RNase 7 Contributes to the Cutaneous Defense against *Enterococcus faecium*

**DOI:** 10.1371/journal.pone.0006424

**Published:** 2009-07-29

**Authors:** Bente Köten, Maren Simanski, Regine Gläser, Rainer Podschun, Jens-Michael Schröder, Jürgen Harder

**Affiliations:** 1 Department of Dermatology, University Hospital Schleswig-Holstein, Campus Kiel, Kiel, Germany; 2 Institute for Infection Medicine, University Hospital Schleswig-Holstein, Campus Kiel, Kiel, Germany; Columbia University, United States of America

## Abstract

**Background:**

Human skin is able to mount a fast response against invading microorganisms by the release of antimicrobial proteins such as the ribonuclease RNase 7. Because RNase 7 exhibits high activity against Enterococcus faecium the aim of this study was to further explore the role of RNase 7 in the cutaneous innate defense system against E. faecium.

**Methodology/Principal Findings:**

Absolute quantification using real-time PCR and ELISA revealed that primary keratinocytes expressed high levels of RNase 7. Immunohistochemistry showed RNase 7 expression in all epidermal layers of the skin with an intensification in the upper more differentiated layers. Furthermore, RNase 7 was secreted by keratinocytes in vitro and in vivo in a site-dependent way. RNase 7 was still active against E. faecium at low pH (5.5) or high NaCl (150 mM) concentration and the bactericidal activity of RNase 7 against E. faecium required no ribonuclease activity as shown by recombinant RNase 7 lacking enzymatic activity. To further explore the role of RNase 7 in cutaneous defense against E. faecium, we investigated whether RNase 7 contributes to the E. faecium killing activity of skin extracts derived from stratum corneum. Treatment of the skin extract with an RNase 7 specific antibody, which neutralizes the antimicrobial activity of RNase 7, diminished its E. faecium killing activity.

**Conclusions/Significance:**

Our data indicate that RNase 7 contributes to the E. faecium-killing activity of skin extracts and suggest an important role for RNase 7 in the protection of human skin against E. faecium colonization.

## Introduction

Human skin is continuously exposed to a wide variety of potential pathogenic bacteria. Despite these threats, human skin is normally not infected. In the last decade it has become evident that human skin provides, in addition to its physical barrier, also a chemical barrier based on the release of antimicrobial proteins [1]–[3]. Antimicrobial proteins are endogenous, gene-encoded proteins, which are able to kill bacteria, fungi and viruses at micro- and nanomolar concentrations. Recent *in vivo* studies confirmed the hypothesis that antimicrobial proteins have the capacity to protect the host against pathogenic microorganisms [4]–[6]. Some of these antimicrobial proteins are upregulated at sites of infection and inflammation such as the human beta-defensins −2 and −3 (hBD-2, hBD-3) as well as the cathelicidin LL-37 [3], [7]–[9]. Upregulation of hBD-2, hBD-3 and LL-37 in the skin provides a rapid first-line of cutaneous defense against invading microorganisms [10]–[17]. Other antimicrobial proteins such as psoriasin (S100 A7) are also expressed in high amounts in healthy skin [18]. Psoriasin is mainly active against the gram-negative bacterium *E. coli* and we recently presented evidence that psoriasin is key for the resistance of human skin against colonization by the gram-negative gut bacterium *E. coli* [18].

Another antimicrobial protein expressed in healthy skin is RNase 7. RNase 7 is a member of the RNase A superfamily that is characterized by homology with bovine ribonuclease A [19]. Members of this family share a conserved structure of six or eight cysteines linked by disulfide bonds and two histidines and one lysine that form the catalytic site [19]. To date, eight human members (RNase 1–8) of the RNase A superfamily have been described. Moreover, five additional genes in the human genome that are related to the RNase A ribonucleases have also been identified (RNase 9–13) [19]. However, the physiological role of these ribonucleases is still not well understood. Recent data suggest that ribonucleases may also play a role in host defense. Eosinophil-derived neurotoxin (EDN; RNase 2) and eosinophil cationic protein (ECP; RNase 3) are localized to eosinophil secretory granules and exhibit antiviral activities [20], [21]. In addition, ECP displays *in vitro* killing activity against various gram-negative and gram-positive bacteria [22]. Antimicrobial activity was also reported for RNase 5 (Angiogenin) [23], a protein which was originally identified from its capacity to induce blood vessel growth [24]. RNase 7 was originally isolated from stratum corneum extracts and cloned from keratinocytes [25]. It exhibited a broad-spectrum of antimicrobial activity [25]. Zhang *et al*. isolated the RNase 7 gene by a genomic database search [26]. RNase 7 mRNA expression was detected in primary keratinocytes and expression was induced by contact with heat-killed bacteria and UV-B radiation [25], [27]. Recently, induction of RNase 7 mRNA expression has been reported in skin biopsies of psoriasis and atopic dermatitis patients [28].

The aim of this study was to further assess the role of RNase 7 in cutaneous defense. A detailed analysis of its *in vitro* as well as *in vivo* expression together with functional antimicrobial studies suggest that RNase 7 may play a major role in skin defense and contributes to the high resistance of human skin against colonization with the gram-positive gut bacterium *E. faecium*.

## Results

### Generation of RNase 7-specific antibodies

**Figure 1:**
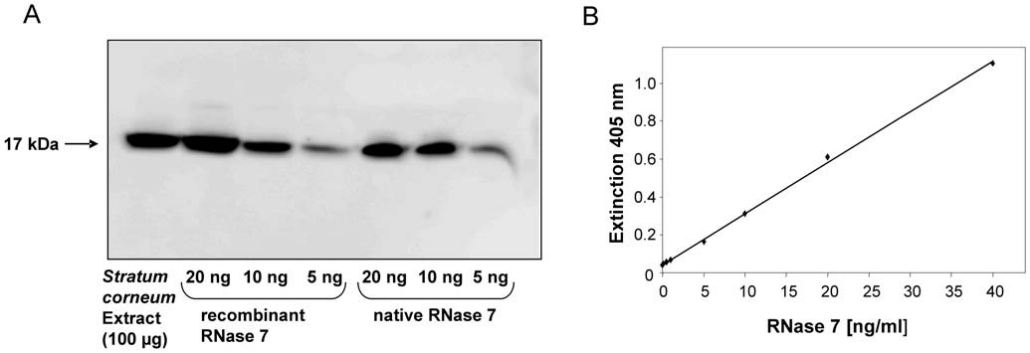
Generation of RNase 7 specific antibodies and ELISA. (A) A goat was immunized with a combination of natural and recombinant RNase 7, and serum was purified with an RNase 7 affinity column. The specificity of the RNase 7 affinity-purified polyclonal antibodies was verified by Western-Blot analysis. 100 µg of stratum corneum extract and different amounts of natural skin-derived RNase 7 and recombinant RNase 7 were subjected to Western-Blot analysis using the affinity-purified polyclonal antibodies raised against RNase 7. (B) The RNase 7 antibodies were used to establish an ELISA as described in the experimental procedures. A representative standard curve of the RNase 7 ELISA is shown. The detection limit of the ELISA was a concentration of 0.3 ng⋅ml^−1^ RNase 7.

First, we successfully expressed RNase 7 in recombinant form in *E. coli*. The serum of a goat immunized by a mixture of natural and recombinant RNase 7 showed high RNase 7 immunoreactivity. Purification of RNase 7 antibodies from the serum using an RNase 7 affinity column led to the isolation of RNase 7 specific antibodies. These antibodies specifically detected RNase 7 in stratum corneum extracts (Fig. 1A). For quantitative analyses and to determine how RNase 7 is secreted *in vivo* at different skin sites, we developed an RNase 7-specific enzyme-linked immunosorbent assay (ELISA) using the RNase 7-specific polyclonal antibodies. Fig. 1B shows a representative standard curve using different concentrations of RNase 7. The detection limit of the ELISA was at a concentration of 0.3 ng⋅ml^−1^. The specificity of the RNase 7 antibodies was further verified by testing other cationic antimicrobial proteins such as lysozyme, hBD-2, hBD-3 and the closely related (78% identity) RNase 8 [29]. All these proteins were not detected by the RNase 7 ELISA (not shown).

### Primary keratinocytes express high levels of RNase 7 mRNA

**Figure 2:**
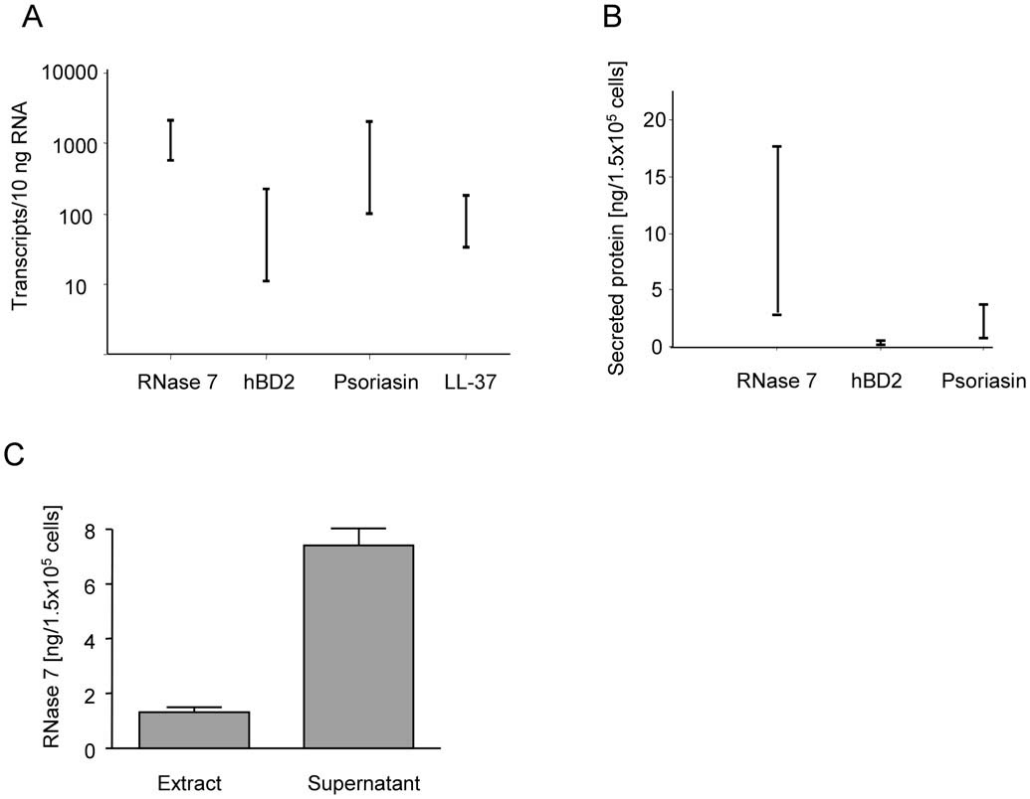
Abundant expression and secretion of RNase 7 in primary keratinocytes. (A) Transcript levels quantified by real-time PCR of various antimicrobial proteins expressed in primary keratinocytes. Shown is the range of data obtained in three independent experiments measured in triplicate. (B) Secretion of antimicrobial proteins from keratinocytes cultured for 16 h. Supernatants of primary keratinocytes were analyzed for RNase 7, psoriasin and hBD-2 by ELISA. Shown is the range of data obtained by three independent experiments measured in triplicate. (C) Comparison of RNase 7 protein present in cell extracts with supernatants from primary keratinocytes cultured for 16 h. Shown are the results of three independent cell culture samples (columns represent means±S.D.).

We used real-time PCR to determine the absolute transcript levels in primary keratinocytes. This quantitative analysis revealed that primary keratinocytes expressed high levels of RNase 7 mRNA when compared to other skin-derived antimicrobial proteins such as human beta-defensin-2 (hBD-2), psoriasin and LL-37 (Fig. 2A).

### RNase 7 is secreted *in vitro* and *in vivo*

**Figure 3:**
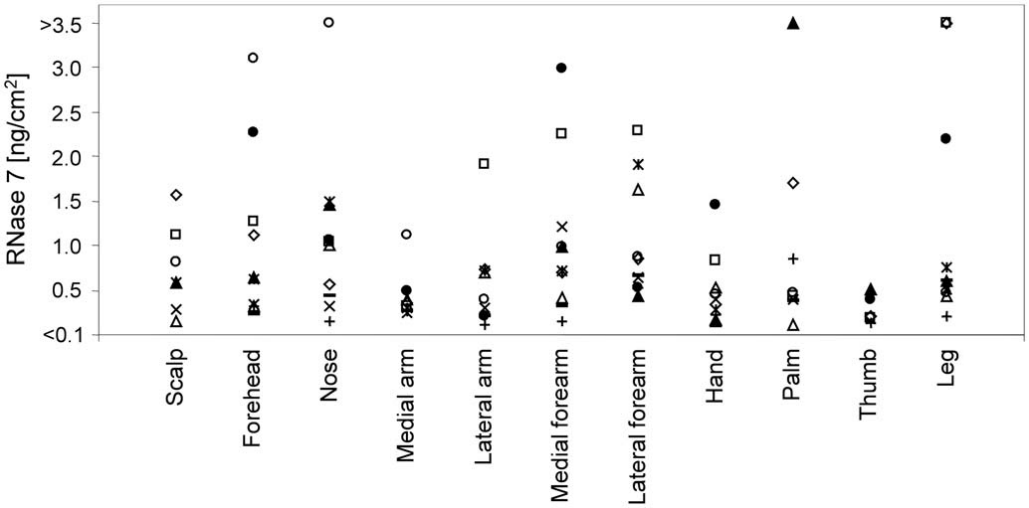
RNase 7 is secreted *in vivo* on the body surface. Standardized areas of various body locations on healthy volunteers (n = 10) were rinsed with 10 mM sodium phosphate buffer containing 150 mM NaCl, pH 7.4 to determine the concentration of RNase 7 at the skin surface by ELISA. Data represent the amount of soluble RNase7/cm^2^. Each symbol represents data from a single volunteer.

To study whether RNase 7 is secreted by keratinocytes, we analyzed keratinocyte culture supernatants as well as cell extracts by ELISA. High amounts (3–18 ng/1.5×10^5^ cells) of RNase 7 were detected in the supernatants of primary keratinocytes cultured for 16 h indicating that RNase 7 is efficiently secreted by the keratinocytes (Fig. 2B). A comparison of RNase 7 amounts present in the culture supernatants and cell extracts derived from primary keratinocytes revealed that the main portion of RNase 7 is released (Fig. 2C). To determine whether RNase 7 is secreted *in vivo*, we investigated the presence of RNase 7 at various skin surface sites. We rinsed standardized 0.5-cm^2^ skin areas of healthy human donors with 500 µl 10 mM sodium phosphate buffer containing 150 mM NaCl, pH 7.4 and analyzed the washing fluids for their content of RNase 7 by ELISA. RNase 7 amounts at skin surfaces depended on the donor (n = 10), the skin area, and previous washings for body care. RNase 7 amounts were mainly in the range between 0.3 and 3 ng⋅cm^−2^ (Fig. 3). In addition, we investigated the concentration of RNase 7 in perianal swabs derived from six individuals. In all six samples RNase 7 was detectable (range of 3.9–12.2 ng⋅ml^−1^; not shown).

### RNase 7 is expressed in all epidermal layers of human skin

**Figure 4:**
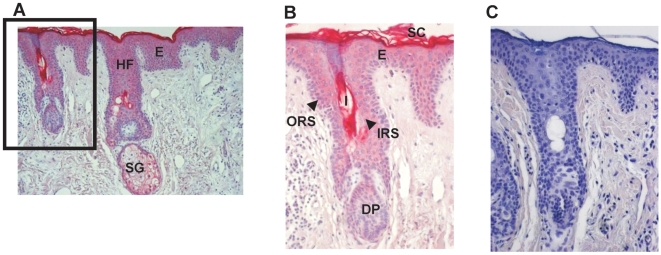
Epidermal keratinocytes express RNase 7 *in vivo*. (A & B) Immunostaining of RNase 7 expression in human normal skin using affinity-purified RNase 7 antibodies. Strong RNase 7 immunoreactivity was detected in the epidermis with highest activity in the uppermost epidermal layers. Sebaecous glands and hair follicles also stained positively. (A) 10×magnification, (B) 20 X magnification of the indicated area of panel A. (C) Negative control using preimmune serum. (E: Epidermis, SC: Stratum Corneum, HF: Hair Follicle, I: Infundibulum, SG: Sebaceous Gland, DP: Dermal Papilla, ORS: Outer Root Sheath, IRS: Inner Root Sheath)

To investigate the distribution of RNase 7 in healthy skin *in vivo*, we performed immunohistochemistry using the RNase 7-specific antibodies. Intense RNase 7 immunoreactivity was present in all epidermal layers of the skin with an intensification of the upper more differentiated layers, especially within stratum corneum. Sebaeceous glands and hair follicles also stained positively. The outer root sheath of hair follicles showed more intensive immunoreactivity when compared to the inner root sheath (Fig. 4).

### Characterization of the antimicrobial activity of RNase 7 against *E. faecium*

**Figure 5:**
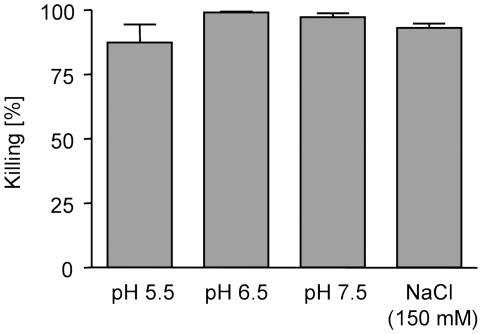
Antimicrobial activity of RNase 7 against *E. faecium* at various pH and high salt conditions. The antimicrobial activity of RNase 7 (1.6 µg⋅ml^−1^) was tested in a microdilution assay against *E. faecium* (ATCC 6057) at various pH conditions or in the presence of 150 mM NaCl. Results are from triplicate determinations and presented as the mean±S.D.

To verify the antimicrobial activity of RNase 7 against *E. faecium*, we investigated natural RNase 7 for microbicidal activity in an antimicrobial microdilution assay against *E. faecium* (ATCC 6057). This strain was effectively killed by RNase 7 (lethal dose of 90% (LD_90_) = 0.4–0.8 µg⋅ml^−1^; not shown). To further support the idea of an antimicrobial function for RNase 7 in cutaneous defense, we performed antimicrobial assays in buffers with variable pH (5.5, 6.5 and 7.4). RNase 7 was active under all pH conditions (Fig. 5). In addition, *E. faecium* was also effectively killed by RNase 7 at 150 mM NaCl (Fig. 5).

### The activity of RNase 7 against *E. faecium* requires no enzymatic activity

**Figure 6:**
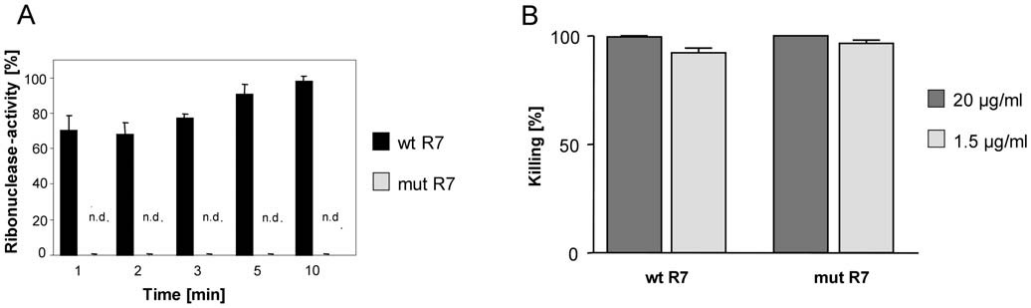
Bactericidal activity of RNase 7 requires no enzymatic activity. (A) Shown are the ribonuclease activities of wildtype non-mutated recombinant RNase 7 (wt R7) and mutated recombinant RNase 7 containing the double mutation, histidin-123 to aspartate and lysine-38 to arginine (mut R7). n.d. = not detectable. (B) The antimicrobial activity of wildtype recombinant RNase 7 (wt R7) and ribonuclease-deficient RNase 7 (mut R7) were tested at concentrations of 20 and 1.5 µg⋅ml^−1^ against *E. faecium* (ATCC 6057). Data are means±S.D.

To assess whether the ribonuclease activity of RNase 7 might be responsible for its antibacterial activity, we expressed recombinant RNase 7 containing two mutated amino acids in its active site required for enzymatic activity. As shown in Fig. 6A, mutated RNase 7 exhibited no ribonuclease activity whereas the recombinant wildtype RNase 7 showed high ribonuclease activity. However, when tested against *E. faecium*, no differences in the killing activity of wildtype and mutated RNase 7 were observed, suggesting that the ribonuclease activity of RNase 7 is not necessary for its antibacterial activity (Fig. 6B). Similar results were obtained with *E. coli* (not shown).

### RNase 7 contributes to the killing activity of human stratum corneum extracts against E. faecium and faecalis

**Figure 7:**
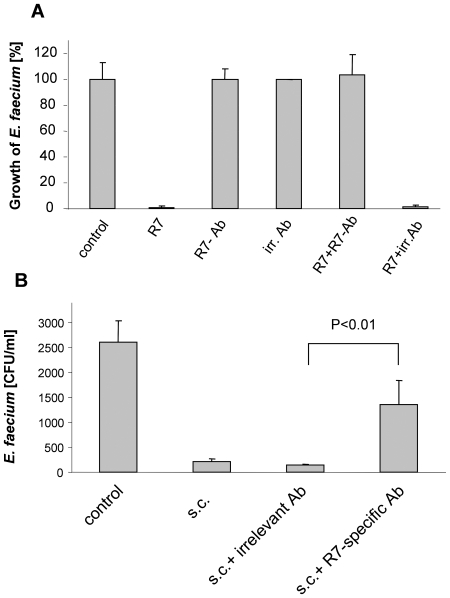
RNase 7 contributes to the killing activity of human stratum corneum extracts against *E. faecium*. (A) RNase 7 (12.5 µg⋅ml^−1^) was tested in a microdilution assay against *E. faecium* (ATCC 6057) alone (R7) or in the presence of 10 mg⋅ml^−1^ RNase 7 antibodies (R7+R7-Ab). Application of the RNase 7 antibodies completely blocked the *E. faecium*-killing activity of RNase 7. As a control, RNase 7 was incubated with irrelevant goat antibodies (R7+irr.Ab). Both, RNase 7 antibodies (R7-Ab) alone as well as irrelevant antibodies (irr. Ab) alone did not influence the growth of *E. faecium*. (B) The killing activity of skin-extracts derived from stratum corneum against *E. faecium* was tested. 2 h incubation of *E. faecium* with stratum corneum extract revealed high killing activity of the extract against *E. faecium* (s.c.). In contrast, the application of RNase 7-blocking antibodies to the stratum corneum extract significantly reduced the killing activity of the extract (s.c.+ R7-specific Ab; p<0.01, Student's *t*-test). Incubation of the stratum corneum extract with the irrelevant antibodies did not affect the killing activity of the extracts (s.c.+ irrelevant Ab). Data show means±S.D. of triplicate samples. A representative result of three independent experiments is shown.

To analyze whether RNase 7 contributes to the *Enterococcus*–killing activity of healthy skin, we determined whether antibodies to RNase 7 affected the killing of *E. faecium* in skin extracts derived from stratum corneum. First, we analyzed whether RNase 7 antibodies neutralized the antibacterial activity of RNase 7 against *E. faecium*. For this purpose we tested the activity of RNase 7 against *E. faecium* in an antibacterial microdilution assay in the presence of RNase 7 antibodies. Fig. 7A shows that application of the RNase 7 antibodies completely blocked the *E. faecium*-killing activity of RNase 7. Antimicrobial activity was not inhibited when equivalent concentrations of irrelevant antibodies (antibodies derived from goat preimmune serum) were used. Having established that the RNase 7-specific antibodies neutralized the antimicrobial effect of RNase 7, we used this approach to investigate the role of RNase 7 for the killing activity of human skin extracts. The application of the RNase 7-blocking antibodies to skin extracts derived from stratum corneum before inoculation with *E. faecium* resulted in a substantial increase in *E. faecium* growth (Fig. 7B).

## Discussion

There is increasing evidence that the skin and other epithelial layers are protected from infection by the release of antimicrobial proteins. Many studies indicate that antimicrobial proteins, like the human beta-defensins, cathelicidin LL-37 and S100 protein psoriasin (S100A7), play an important role in cutaneous defense [1], [3]. Although several reports suggest that the human RNase A superfamily members ECP (eosinophil cationic protein, RNase 3) and EDN (eosinophil-derived neurotoxin, RNase 2) are important effector molecules of eosinophilic granulocytes [30], [31], the role of RNases in epithelial defense is largely unknown. A recent study indicates that a mouse ribonuclease (angiogenin 4) may participate in regulating the intestinal microflora [23].

The recent isolation of the antimicrobial ribonuclease RNase 7 from stratum corneum of healthy skin prompted us to further analyze its role in cutaneous defense. A quantitative analysis revealed that primary keratinocytes express high levels of RNase 7 mRNA when compared to other skin-derived antimicrobial proteins such as psoriasin, hBD-2, and LL-37. This observation further strengthened the hypothesis that RNase 7 may be an important defense molecule in cutaneous innate immunity. To get further insight into RNase 7 protein expression, we generated RNase 7 specific antibodies. Immunohistochemistry using these novel antibodies revealed expression of RNase 7 in all layers of human epidermis with higher intensity in the more outer differentiated epithelial layers. Hair follicles also stained positively which is in concordance with a recent study demonstrating RNase 7 expression in hair follicle epithelium [32]. The expression of RNase 7 in the uppermost epidermal layers and its expression in hair follicles are in concordance with its proposed antimicrobial function, because it is expressed at areas where the first contact with bacteria takes place. Because the deduced RNase 7 precursor contains a signal sequence, we hypothesized that RNase 7 may be efficiently secreted by keratinocytes. Using an RNase 7-ELISA, that we developed, we were able to detect higher RNase 7 protein levels in the supernatants of the primary keratinocyte cultures compared with keratinocyte extracts. These data indicate that RNase 7 is secreted from viable cells and acts primarily outside the cell, which is consistent with the proposed role of RNase 7 in antimicrobial defense.

Based on these *in vitro* data, we speculated that RNase 7 is secreted *in vivo* on the body surface. To prove this hypothesis, we determined whether RNase 7 is present at various skin surface sites. We were able to recover various amounts of RNase 7 in skin washing fluids from different body sites, which confirmed that RNase 7 is secreted *in vivo*. High variability in RNase 7 secretion levels was detected in different persons and skin locations. This suggests that levels of RNase 7 secretion may depend on environmental factors such as microbial colonization.

We recently identified psoriasin as a principal *E. coli*–killing factor that protects the skin from infections with the gut bacterium *E. coli* [18]. However, the high expression of RNase 7 in skin and its very potent activity against *E. faecium* [25] suggest that the skin is also protected against colonization with *E. faecium*. This would explain the low infection rate of skin areas exposed to *E. faecium* such as the perianal region. To prove this hypothesis, we first explored whether the skin has the capacity to kill *E. faecium*. Skin extracts derived from the stratum corneum efficiently killed *E. faecium* indicating that the skin harbors defense mechanisms which inhibit cutaneous colonization with *E. faecium*. To further evaluate whether RNase 7 might contribute to these defense mechanisms, we specifically inhibited the antimicrobial activity of RNase 7 in the skin extracts using RNase 7-specific antibodies. These experiments revealed that RNase 7 contributes to the *E. faecium* killing activity of skin extracts, suggesting an important role for RNase 7 in protecting the skin against *E. faecium* infection.

The high antimicrobial activity of RNase 7 against *E. faecium* raises the question of the responsible molecular killing mechanisms. We speculated that the high ribonuclease activity of RNase 7 might be involved in the RNase 7-mediated killing of *E. faecium*. To address this question, we generated recombinant RNase 7 that lacks enzymatic activity. By making point mutations of the catalytic residues lys-38 and his-123, we found that ablation of ribonuclease activity had no impact on the bactericidal activity of RNase 7 against *E. faecium*. This is consistent with other reports showing that members of the RNase A superfamily exhibit ribonuclease-independent antibacterial activity [33]–[35], but raises the question of the role of the enzymatic activity. It has been shown that the antiviral activity of the RNases ECP and EDN against respiratory syncytial virus (RSV) requires functional ribonuclease activity [20], [21]. These data suggest that the ribonuclease activity of RNase 7 may be necessary for a potential antiviral rather than antibacterial activity. However, this remains to be proven as it is not yet known whether RNase 7 exhibits antiviral activity.

Recently, it has been reported that the ribonuclease inhibitor interacts with RNase 7 and blocks its ribonuclease as well as antimicrobial activity [36]. It is possible that the ribonuclease inhibitor masks the amino acid residues responsible for the antimicrobial action of RNase 7. Another explanation is that the interaction of the ribonuclease inhibitor RI induces a conformational change of RNase 7 as recently reported for the interaction of RNase 1 and the ribonuclease inhibitor [37].

RNase 7 is a highly cationic protein with a pI of approximately 10.7. Many human antimicrobial proteins are cationic and contain a large number of basic amino acids. Examples comprise the alpha- and beta-defensins, the cathelicidins as well as the histatins [7], [38], [39]. It is believed that the highly cationic character of these molecules results in a high affinity to the negatively charged surface of bacteria, which is prerequisite for efficient killing [40]. Very recently, Huang and colleagues showed by NMR and mutagenesis studies that cationic lysines are critical for the antimicrobial activity of RNase 7 against *P. aeruginosa*. They suggested that RNase 7 may bind to the negatively charged components of the bacterial membrane through the presence of cationic residues which ultimately leads to membrane disruption [34]. It remains to be shown whether this killing mechanism, which has been proposed for many antimicrobial peptides [40], is also responsible for the high killing activity of RNase 7 against *E. faecium*.

In summary, our data suggest that RNase 7 contributes to cutaneous innate immunity against *E. faecium* and probably other bacteria. An interesting speculation is that dysregulation of RNase 7 may result in higher susceptibility to infectious diseases. A better understanding of the role of endogenous antimicrobial proteins such as RNase 7 may result in the development of novel therapeutic strategies that enhance the cutaneous innate defense system by the application or selective induction of antimicrobial proteins.

## Materials and Methods

### Ethics Statement

All experiments were performed according to the Declaration of Helsinki protocols and under protocols approved by the Ethics Committee at the Medical Faculty of the Christian-Albrechts-University, Kiel, Germany (A104/06).

### Culture of epithelial cells

Keratinocytes were derived from foreskin samples obtained from circumcision surgery after obtaining written informed consent. The protocol was approved by the Ethics Committee at the Medical Faculty of the Christian-Albrechts-University, Kiel, Germany. Foreskin-derived primary keratinocytes were isolated from foreskins as described [41] and cultured in Epilife-medium (Sigma) in a humidified atmosphere with 5% CO_2_. For stimulation experiments, cells were seeded in 12-well tissue culture plates (3.8 cm^2^⋅well^−1^, BD Biosciences) and used at 60-80% confluence.

### RNA isolation and cDNA synthesis

After treatment, cells were washed twice with PBS and harvested using TRIzol reagent (Invitrogen, San Diego, CA) according to the supplier's protocol. RNA quality and quantity were determined by gel electrophoresis and photometry. Subsequently, 1 µg of total RNA was reverse transcribed to cDNA with oligo dT- primers and 50 Units Superscript II (Invitrogen) according to the manufacturer's protocol.

### Real-time PCR

Real-time PCR analyses were performed in a fluorescence thermal cycler (LightCycler; Roche Diagnostics GMBH). cDNA corresponding to 10 ng RNA served as a template in a 10 µl reaction containing 0.5 µM of each primer and 1 x SYBR *Premix Ex Taq* mix (TaKaRa). Samples were loaded into capillary tubes and incubated in the fluorescence thermal cycler (LightCycler) for an initial denaturation at 95°C for 10 min followed by 45 cycles, each cycle consisting of 95°C for 15 s, 60°C (touchdown of −1°C⋅cycle^−1^ from 66°C to 60°C) for 5 s and 72°C for 10 s. At the end of each run, melting curve profiles were produced by cooling the sample to 65°C for 15 s and then heating slowly at 0.20°C⋅s^−1^ up to 95°C with continuous measurement of fluorescence to confirm amplification of specific transcripts. Cycle-to-cycle fluorescence emission readings were monitored and analyzed using LightCycler Software (Roche Diagnostics GMBH). The specificity of the amplification products was further verified by subjecting the amplification products to electrophoresis on a 2% agarose gel. The fragments were visualized by ethidium bromide staining and the specificity of PCR products was verified by sequencing of representative samples. The following intron spanning primers were used: RNase-7 5′- GGA GTC ACA GCA CGA AGA CCA -3′ (forward primer) and 5′- CAT GGC TGA GTT GCA TGC TTG A -3′ (reverse primer); hBD-2 5′- GCC TCT TCC AGG TGT TTT TG -3′ (forward primer) and 5′- GAG ACC ACA GGT GCC AAT TT -3′ (reverse primer); psoriasin 5′- AGA CGT GAT GAC AAG ATT GAC -3′ (forward primer and )5′- TGT CCT TTT TCT CAA AGA CGT C -3′ (reverse primer); LL-37 5′-GGG GCT CCT TTG ACA TCA GT -3′ (forward primer) and 5′- TGG GTA CAA GAT TCC GCA AA -3′ (reverse primer). Standard curves were obtained for each primer set with serial dilutions of plasmids containing the amplification product. Absolute transcript levels are shown per 10 ng total RNA.

### Expression of recombinant RNase 7 in *E. coli*

The cDNA encoding the 128 amino acids containing natural form of RNase 7 was cloned into the expression vector pET-32a (Novagen), which contains an N-terminal His-Tag sequence allowing purification of the fusion-protein by the use of a nickel-affinity column. A 200 ml culture of transformed *E. coli* (strain BL21pLysS, Novagen) was grown to an optical density of 0.6 and expression was induced by adding 1 mM IPTG. Expression was carried out for 3 h and bacteria were harvested by centrifugation at 6000 x g for 5 min and lysed by sonication. Extracts were purified with a nickel-affinity column (Macherey-Nagel) followed by C8 reversed-phase HPLC as described for the purification of human beta-defensin-3 [42]. The N-terminal part of the purified fusion protein was cleaved off by enterokinase (Invitrogen) and the resulting mature 128 amino acids containing RNase 7 protein was purified by C2/C18 reversed phase HPLC as previously described [42]. Mass analysis using electrospray ionization mass spectrometry (QTOF-II Hybrid-mass spectrometer; Micromass) yielded a mass of 14,546 Da, which exactly corresponds to the mass of natural RNase 7 [25].

Recombinant RNase 7 without ribonuclease activity was generated by site-directed mutagenesis to introduce mutations in the active center of RNase 7. First, histidin at position 123 was mutated to aspartate using the following primer: sense 5′- ACT GAG ATC TGG GTA CCG ACG ACG ACG ACA AGA AGC CCA AGG GCA TGA CCT C -3′, antisense 5′- ATT TGC GGC CGC CTA AAG GAC TCT GTC CAA GTC TAC AGG -3′. The resulting PCR product was digested with the restriction enzymes *NOT*I and *Bgl*II and subsequently cloned into the expression vector pET-32a. This plasmid was used as a template to mutate lysine at position 38 to arginine using the “QuikChange Multi Site-Directed Mutagenesis Kit” (Stratagene) and the following primers: 5′- GCA CAC AAA ACG GTG CAG AGA CCT CAA CAC C 3′, antisense 5′- GGT GTT GAG GTC TCT GCA CCG TTT TGT GTG C- 3′. The mutated plasmids were used for expression in *E. coli* as described above. The correct mass of the resulting protein was verified by electrospray ionization mass spectrometry.

### Generation of RNase 7-specific antibodies

0.5 mg natural skin-derived RNase 7 was mixed with 1.3 mg recombinant RNase 7 and the resulting 1.81mg were used for immunization. 1.2 mg of this RNase 7 preparation was conjugated to keyhole limpet hemocyanine (KLH, Sigma) using glutaraldehyde. Therefore, 1 mg KLH in 1 ml PBS was mixed with 1 µl 25% glutaraldehyde (Serva) and incubated for 1 h at room temperature with gently shaking. After incubation, the reaction mixture was diafiltrated and concentrated in 400 µl PBS using a vivaspin 0.5 ml concentrator column (30 kDa cut off, Vivascience). The KLH-glutaraldehyde concentrate (400 µl) was incubated with 600 µl of 1.2 mg RNase 7 in PBS for 1 h at room temperature with gentle rotation. The reaction was stopped by the addition of 5 µl 1 M Tris (pH 8.0). 500 µl of 0.6 mg RNase 7 in PBS was added and the preparation was divided into one 450 µl aliquot for initial immunization and three 350 µl aliquots for booster immunization of a goat. The immunization was carried out by the “ZIKA-Kaninchenbetrieb” (Gottin, Germany). We generated an RNase 7 affinity column to selectively isolate RNase 7 specific antibodies from the serum. Therefore, 1.5 mg of recombinant RNase 7 was coupled to a 1 ml HiTrap NHS-activated column (Amersham Pharmacia Biotech) according to the manufacturer's protocol. 500 µl of goat anti-RNase 7 serum was loaded onto the affinity column and the column was washed with 10 mM sodium phosphate buffer, pH 7.3. Low-affinity antibodies were eluted using 1 M NaCl (pH 7) and high-affinity RNase 7 antibodies were eluted using 200 mM glycine (pH 3), immediately neutralized with 1 M Tris (pH 7.5) and dialfiltered against PBS.

### ELISA

96-well immunoplates (MaxiSorp™, Nunc) were coated at 37°C for 1 h with 50 µl affinity-purified goat anti-RNase 7 antibody diluted 1∶1000 to 1 µg⋅ml^−1^ in 0.05 M carbonate buffer, pH 9.6. Subsequently, wells were blocked with 200 µl 1% bovine serum albumin in PBS for 10 min at room temperature. After washing three times with 200 µl PBS+0.1% Tween 20, 50 µl per well of cell culture supernatants and serial dilutions of natural skin-derived RNase 7 in cell culture medium were incubated for 30 min at room temperature. Plates were washed three times with PBS+0.1% Tween 20 and wells were incubated for 30 min at room temperature with 50 µl of biotinylated goat anti-RNase 7 antibody diluted 1∶7500 to 1.3 µg⋅ml^−1^ in PBS+0.1% Tween 20. Plates were washed again three times with PBS+0.1% Tween and filled with 50 µl⋅well^−1^ of Streptavidin-POD (Roche Diagnostics; 1∶10000 in PBS+0.1% Tween 20). The plates were then incubated for 30 min at room temperature, washed six times as described above, and incubated with the development agent 2,2′-azino-bis-3-ethylbenzthiazoline-6-sulfonic acid (ABTS; Roche Diagnostics) for 15 min at room temperature in the dark. Absorbance was measured at 405 nm with a multichannel photometer (Sunrise; Tecan, Crailsheim, Germany). The psoriasin and hBD-2 ELISAs were performed as previously described [18], [43].

### Western blot

Stratum corneum derived from the heel of healthy individuals was extracted with acidic ethanolic citrate buffer as previously described [44]. Stratum corneum extract was mixed with 4x NuPAGE LDS Sample Buffer (Invitrogen) and 0.5 µg of this extract was separated onto a NuPAGE 4-12% Bis-Tris Gel (Invitrogen). Proteins were transferred to a Protran-nitrocellulose membrane (Schleicher & Schuell BioScience), blocked for 1 h in blocking buffer (5% (w⋅v^−1^) nonfat powdered milk in PBS+0.05% Tween), then incubated for 18 h at 4°C in 3% (w⋅v^−1^) nonfat powdered milk in PBS+0.05% Tween containing RNase 7 affinity-purified antibody (10 µg⋅ml^−1^). The membrane was washed with PBS+0.05% Tween six times for 5 min each, then incubated for 1 h in 3% (w⋅v^−1^) nonfat powdered milk in PBS+0.05% Tween containing 1∶20000 dilution of mouse anti-goat IgG HRP conjugate (Dianova). After another six washes, the membrane was incubated for 5 min with chemiluminescent peroxidase substrate (Sigma) and visualized using a Diana III cooled CCD-camera imaging system (Raytest, Straubenhardt, Germany).

### Immunohistochemistry

To localize the protein expression of RNase 7 in human skin biopsies, immunhistochemical staining was performed. Therefore 5 µm vertical paraffin sections were deparaffinised and rehydrated followed by heat-induced antigen retrieval in 0.01 M citrate buffer (pH 6.0). The slides were blocked with normal rabbit serum (1∶5, Dako Cytomation) and incubated for 60 min with the self-generated polyclonal goat anti-RNase 7 antibody (10 mg⋅ml^−1^, 1∶400). Subsequently, the sections were incubated with a biotinylated rabbit anti-goat IgG antibody (1∶500, Dianova) followed by incubation with Vector Universal ABC Alkaline Phophatase Substrate Kit (Vector), development with Vector NovaRED Substrate (Vector) and counterstaining with hematoxylin. Negative control straining was performed by using preimmune serum.

### Antimicrobial and ribonuclease assay

Natural skin-derived RNase 7 was purified from skin extracts as previously described [25] and used for a standard antimicrobial microdilution assay as described previously [29]. Briefly, test organisms were incubated with various concentrations of RNase 7 in 10 mM sodium phosphate buffer (pH 7.4 or the indicated pH conditions) containing 1% (v⋅v^−1^) trypticase soy broth for 3 h at 37 °C. The antibiotic activity of RNase 7 was analyzed by plating serial dilutions of the incubation mixtures and determining the number of colony-forming units (CFUs) the following day.

The ribonuclease activity of recombinant RNase 7 was determined against a standard yeast tRNA substrate as described previously [25].

### Antimicrobial activity of stratum corneum extracts

Stratum corneum derived from the heel of healthy individuals was extracted as previously described [44] and diafiltered against 10 mM sodium phosphate buffer (pH 7.4). ELISA analysis revealed concentrations of 7–63 µg RNase 7 per gram stratum corneum. *E. faecium* (ATCC 6057; 4×10^3^/ml) was incubated with 1 µl of stratum corneum in 50 µl 10 mM sodium phosphate buffer (pH 7.4). The same experiment was carried out with application of 10 µl R7 antibody (10 mg⋅ml^−1^) or equivalent concentrations of irrelevant antibodies (antibodies derived from goat preimmune serum). Killing activity was analyzed by plating serial dilutions of the incubation mixture and counting the colony-forming units (CFU) the following day.
